# Artificial intelligence-assisted colonoscopy improves adenoma detection rates in routine colonoscopy practice: a single-center, retrospective, propensity score-matched study with concurrent controls

**DOI:** 10.1186/s12876-025-04011-w

**Published:** 2025-10-23

**Authors:** Da Yeon Ham, Jae Gon Lee, Chung Il Ahn, Sea Hyub Kae, Hyun Joo Jang

**Affiliations:** 1 Division of Gastroenterology, Department of Internal Medicine, Dongtan Sacred Heart Hospital, Hallym University, Hallym University College of Medicine, 7 Keunjaebong-gil, Hwaseong, 18450 Korea; 2 Division of Gastroenterology, Department of Internal Medicine, Hanyang University Guri Hospital, Hanyang University College of Medicine, Guri, Korea; 3 Department of Artificial Intelligence, INFINITT Healthcare Co., Ltd, Seoul, Korea

**Keywords:** Artificial intelligence, Colonoscopy, Colonic polyp, Cancer screening

## Abstract

**Background/Aims:**

This study aimed to investigate whether a real-time artificial intelligence (AI)-assisted polyp detection system can improve adenoma detection rates (ADRs) in real-world colonoscopy practice.

**Methods:**

This single-center, retrospective, propensity score-matched study collected data from consecutive patients who underwent colonoscopy—either AI-assisted or standard colonoscopy— between March 2023 and February 2024. Propensity score matching was conducted to adjust for baseline characteristics across the groups.

**Results:**

During the study period, 1,085 patients who underwent colonoscopy were eligible for inclusion. After propensity score matching, 474 patients who underwent AI-assisted colonoscopy and 474 who underwent standard colonoscopy were included in the primary analysis. The ADR was significantly higher in the AI-assisted colonoscopy group than in the standard colonoscopy group (35.9% vs. 26.4%; *p* = 0.002). Additionally, the number of adenomas detected per colonoscopy was significantly higher in the AI-assisted group than in the standard group (0.69 ± 1.22 vs. 0.43 ± 0.91; *p* < 0.001). However, the detection rates of advanced adenomas and sessile serrated lesions did not differ significantly between the two groups.

**Conclusion:**

AI-assisted colonoscopy significantly improves ADRs in real-world colonoscopy practice.

## Introduction

Colorectal cancer (CRC) ranks as the third most common cancer and is the second leading cause of cancer-related mortality worldwide [1]. Since most CRCs originate from precursor adenomas, colonoscopy and polypectomy can prevent CRC and related deaths [2]. Additionally, the adenoma detection rate (ADR) has long been considered a key quality indicator for screening colonoscopy, as a higher ADR is associated with a reduced risk of CRC [3]. However, ADRs vary widely among endoscopists [4], with over 20% of adenomas missed during colonoscopy [5]. In addition, even the same examiner can experience variations in the ADR of over 10% owing to factors such as time pressure, patient characteristics, sedation status, and examiner’s fatigue [6]. 

Recent advances in artificial intelligence (AI) have led to the introduction of computer-aided detection (CADe) in colonoscopy practice to enhance quality and address operator-dependent limitations. Multiple randomized controlled trials (RCTs) have demonstrated that AI-assisted colonoscopy significantly increases ADRs compared with standard colonoscopy [7–9]. However, several real-world clinical studies failed to demonstrate an increase in ADRs with AI-assisted colonoscopy, suggesting that the benefits reported in RCTs may not be reproducible [10–12]. Moreover, two recent meta-analyses of real-world AI-assisted colonoscopy studies showed mixed results regarding increased ADRs [13, 14]. Therefore, more evidence is required to evaluate the impact of AI-assisted colonoscopy in real-world settings. This study aimed to determine whether AI-assisted colonoscopy can improve ADRs in uncontrolled, real-world clinical practice using a retrospective observational study design with concurrent controls.

## Materials and methods

### Study design and patients

This single-center, retrospective, propensity score-matched study was conducted at Hallym University Dongtan Sacred Heart Hospital. The study protocol was approved by the Institutional Review Board of Hallym University Dongtan Sacred Heart Hospital (approval no. HDT 2023-04-001) and adhered to the ethical guidelines of the Declaration of Helsinki.

All patients who underwent colonoscopy for routine indications between March 2023 and February 2024 were included. Indications for colonoscopy included CRC screening, post-polypectomy surveillance, and diagnostic colonoscopy for symptomatic patients. Exclusion criteria included colonoscopy for resection of known colorectal polyps, poor bowel preparation (Boston Bowel Preparation Scale [BBPS] total score < 6 or any segment < 2; the BBPS is a validated scoring system developed at Boston University School of Medicine to assess bowel cleanliness during colonoscopy [15]. It divides the colon into three segments—right, transverse, and left—and assigns a score from 0 to 3 for each segment based on mucosal visibility, with a total score ranging from 0 to 9), prior bowel resection, inflammatory bowel disease, polyposis syndrome, advanced CRC, failure of cecal intubation, sigmoidoscopy only, inability to resect polyps (e.g., when antiplatelet agents or anticoagulation could not be stopped), age < 20 years, and colonoscopies performed by endoscopists not participating in the study. Data from endoscopists who did not participate in the study (those who performed colonoscopies in only the AI-assisted or conventional endoscopy unit) were excluded to minimize potential bias in the overall ADR outcomes.

### Colonoscopy procedure and data collection

Of the four active endoscopy units, two were equipped with AI-assisted endoscopy systems. Patients were randomly assigned to each endoscopy unit by the nurse in charge according to the routine workflow of the endoscopy department. Therefore, patients assigned to AI-assisted endoscopy units underwent colonoscopy with a real-time AI-based polyp detection system, while those assigned to conventional endoscopy units underwent standard colonoscopy. Four experienced endoscopists (each having performed at least 3000 colonoscopies) and five endoscopy trainees (first-year gastroenterology fellows) performed the colonoscopies. All participants were aware that their colonoscopy performance was being monitored for the study.

All colonoscopies were performed using a video endoscopy system and a high-definition endoscope (Fujifilm ELUXEO 7000 system and 600 series colonoscopes; FUJIFILM, Tokyo, Japan) with standard white-light imaging. Chromoendoscopy and image-enhanced endoscopy were not used in standard inspection for polyp detection but were occasionally used for lesion characterization after polyp detection at the discretion of each endoscopist. Distal attachments, such as transparent caps, were used according to the endoscopist’s preference. All patients who underwent colonoscopy received standard bowel preparation with polyethylene glycol-based or oral sulfate-based preparation regimens. Most patients received standard sedation during endoscopy using available sedative agents (e.g., midazolam and/or propofol); however, some patients opted not to be sedated based on personal preferences or underlying medical conditions. All detected polyps were removed endoscopically unless invasive cancer was suspected, in which case they were histologically evaluated.

Data from all consecutive patients who underwent colonoscopy between March 2023 and February 2024 were collected retrospectively: age, sex, body mass index (BMI), indications for colonoscopy, grade of bowel preparation, inspection time, and characteristics of detected polyps (location, size, morphology, and histologic findings) were recorded on a standardized data collection sheet. Bowel preparation was assessed using the BBPS [16]. Lesion morphology was described according to the Paris classification [17]. All colonoscopies were performed using CO₂ insufflation. The net inspection time was calculated by subtracting the time required for biopsy or polyp resection from the total time spent on the procedure.

### Real-time AI-assisted polyp detection system

**Fig. 1:**
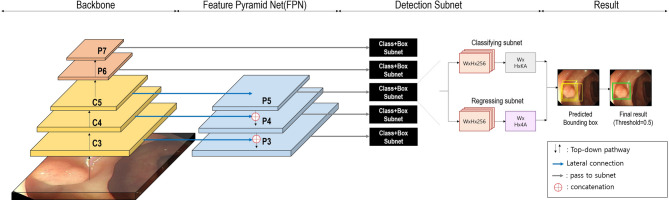
Illustration of proposed SmartEndo-Net architecture for colorectal polyp detection. It consists of a top-down pathway with ResNet-50 and a Feature pyramid network (FPN) with lateral connections from the backbone. For each scale, two subnets are utilized to classify and regress the predicted bounding box. As a result, when the predicted probability of the bounding box exceeds the threshold value of 0.5, both a green bounding box and an alarm sound are activated

The SmartEndo system (INFINITT Healthcare, Seoul, Korea) was used for the study. SmartEndo is a real-time, computer-aided polyp detection system based on a deep-learning algorithm that can be integrated with any endoscopic system. The system automatically detects colorectal polyps during colonoscopy and displays a green box on the screen for identified lesions, accompanied by alarm sounds. The details of the proposed network for automatic polyp detection are shown in Fig. 1. On the backbone of the SmartEndo-Net, to extract the rich information of polyp, ResNet-50 used by residual block is used. To obtain detailed multiscale polyp information, a feature pyramid network (FPN) was used to construct a top-down pathway and lateral connections. After constructing the backbone, two subnets were attached to each scale. The subnets consist of one designed for classifying the predicted bounding box and another for regressing the predicted bounding box to the ground-truth bounding box. Focal loss was applied at the output of the classification subnet to address the imbalance between the foreground and background of the predicted bounding box. Focal loss adjusted the cross-entropy to assign a larger loss to well-classified samples, thereby reducing loss in these samples. Finally, a polyp was predicted when the probability of the bounding box exceeded the threshold value of 0.5. When a polyp is detected in real time, a green bounding box is displayed, and an alarm rings.

### Outcome measures

The primary outcome was the ADR, defined as the percentage of colonoscopies with at least one adenoma among all colonoscopies. Secondary outcomes included lesion detection rates (for advanced adenomas, sessile serrated lesions [SSLs], polyps, and non-neoplastic lesions) and the number of lesions per colonoscopy (including adenomas, advanced adenomas, SSLs, polyps, and non-neoplastic lesions). The detection rates and number of lesions were calculated by the endoscopists. The characteristics of detected polyps in each group were presented through a per-polyp analysis. Advanced adenomas were defined as the presence of any of the following: any adenomas ≥ 10 mm, high-grade dysplasia (or intramucosal carcinoma), and adenomas with a villous component. Non-neoplastic lesions were defined as polyps that are neither adenomas nor SSLs. The predictors of ADR were analyzed using regression analysis.

### Statistical analysis

Propensity score matching analysis was performed using the nearest neighbor method based on age, sex, BMI, indications for colonoscopy, bowel preparation scale, and inspection time. Continuous variables were presented as means ± standard deviations (SD) and compared using the t-test. Categorical variables were presented as numbers (%) and compared using the chi-square test (or Fisher’s exact test, if appropriate). Multivariate logistic regression analysis was performed to identify predictive factors for adenoma detection, with results presented as odds ratios (ORs) and 95% confidence intervals (CIs). A *p*-value of < 0.05 was considered statistically significant. All statistical analyses were conducted using R version 4.4.1 (R Foundation for Statistical Computing, Vienna, Austria).

## Results

### Study flow and baseline characteristics of the patients

**Fig. 2:**
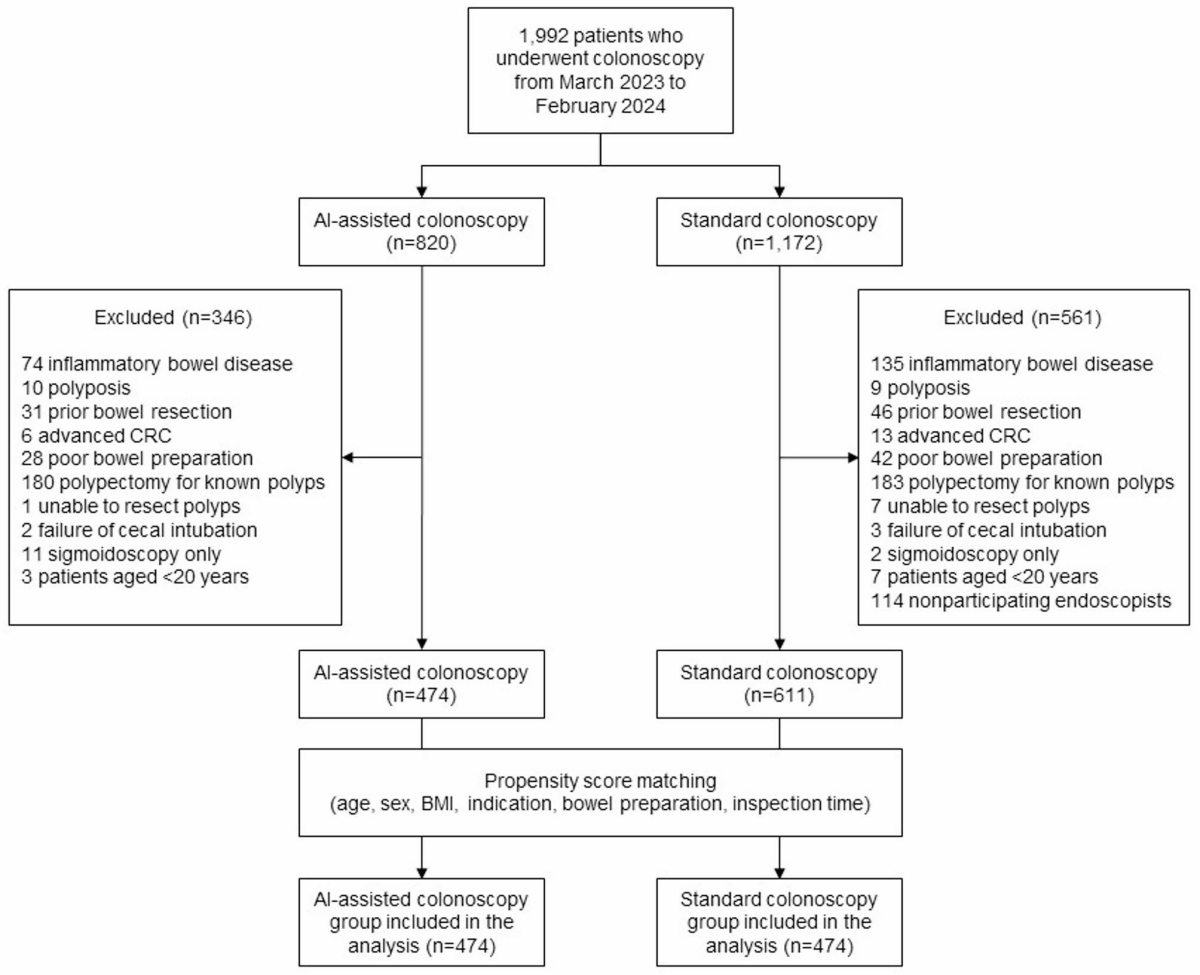
Study flowchart AI, artificial intelligence; CRC, colorectal cancer; BMI, body mass index

A total of 1,992 patients underwent colonoscopy between March 2023 and February 2024. Among these, 820 underwent AI-assisted colonoscopy, while 1,172 underwent standard colonoscopy. After excluding 907 patients based on predefined exclusion criteria, 1,085 patients remained eligible. Propensity score matching was conducted based on age, sex, BMI, indications for colonoscopy, bowel preparation scale score, and inspection time. Consequently, 948 patients (474 in each group) were included in the final analysis. The study flowchart is shown in Fig. 2.

Table 1 shows the baseline characteristics of the patients in each group in the original and propensity score-matched cohorts. In the original cohort, there were significant differences in indications and bowel preparation scales between the two groups. In contrast, there were no significant differences in baseline characteristics between groups in the matched cohort.

**Table 1:** Baseline characteristics of the patients

	Original cohort			Matched cohort		
	AI group (*n* = 474)	SC group (*n* = 611)	*p*-value	AI group (*n* = 474)	SC group (*n* = 474)	*p*-value
Age	55.4 ± 12.9	55.4 ± 13.5	0.977	55.4 ± 12.9	54.9 ± 13.1	0.543
Female sex	222 (46.8)	283 (46.3)	0.914	222 (46.8)	231 (48.7)	0.603
BMI (kg/m^2^)	24.01 ± 3.65	24.08 ± 3.64	0.744	24.01 ± 3.65	24.07 ± 3.70	0.807
Indication			< 0.001			< 0.001
Screening Surveillance Symptomatic	48 (10.1) 317 (66.9) 109 (23.0)	95 (15.5) 335 (54.8) 181 (29.6)		48 (10.1) 317 (66.9) 109 (23.0)	46 ( 9.7) 316 (66.7) 112 (23.6)	
BBPS	8.61 ± 0.75	8.75 ± 0.63	0.001	8.61 ± 0.75	8.70 ± 0.69	0.059
Inspection time (s)	452.8 ± 162.2	440.3 ± 173.2	0.222	452.8 ± 162.2	445.5 ± 176.6	0.505

Values are presented as mean ± standard deviation or numbers (%)

BMI, body mass index; BBPS, Boston Bowel Preparation Scale; AI, AI-assisted colonoscopy; SC, standard colonoscopy

### Lesion detection outcomes

Table 2 summarizes the outcomes of lesion detection by the colonoscopy group. The overall ADR was significantly higher in the AI-assisted colonoscopy group than in the standard colonoscopy group (35.9% vs. 26.4%; *p* = 0.002). The number of adenomas per colonoscopy (APC) was 0.69 ± 1.22 in the AI-assisted colonoscopy group and 0.43 ± 0.91 in the standard colonoscopy group, showing a statistically significant difference (*p* < 0.001). In addition, polyp detection rates (PDRs) and the number of polyps per colonoscopy were significantly higher in the AI-assisted colonoscopy group compared to the standard colonoscopy group (PDR: 53.2% vs. 46.2%, respectively, *p* = 0.038; the number of polyps per colonoscopy: 1.23 ± 1.65 vs. 0.93 ± 1.39, respectively, *p* = 0.002). However, the detection rates and numbers of advanced adenomas, SSLs, and non-neoplastic lesions did not differ significantly between the two groups.

**Table 2:** Lesion detection outcomes according to colonoscopy groups

		AI group (*n* = 474)	SC group (*n* = 474)	*p*-value
Lesion detection rates (%)				
	Adenomas	35.9	26.4	0.002
	Advanced adenomas^*^	3.2	2.1	0.418
	Sessile serrated lesions	5.5	3.0	0.076
	Any polyps	53.2	46.2	0.038
	Non-neoplastic lesions^†^	17.3	18.4	0.734
Number of lesions per colonoscopy (mean ± SD)				
	Adenomas	0.69 ± 1.22	0.43 ± 0.91	< 0.001
	Advanced adenomas	0.04 ± 0.21	0.02 ± 0.16	0.298
	Sessile serrated lesions	0.07 ± 0.32	0.05 ± 0.33	0.276
	Any polyps	1.23 ± 1.65	0.93 ± 1.39	0.002
	Non-neoplastic lesions	0.22 ± 0.56	0.23 ± 0.54	0.858

SD, standard deviation; AI, AI-assisted colonoscopy; SC, standard colonoscopy

^*^Advanced adenomas were defined as adenomas ≥ 10 mm, and/or with villous histology, and/or with high-grade dysplasia

^†^Non-neoplastic lesions were defined as lesions other than adenomas or sessile serrated lesions

### Characteristics of the detected lesions

A total of 1,175 polyps were detected in the full cohort (*n* = 1,085), of which 583 were detected in the AI-assisted colonoscopy group and 592 in the standard colonoscopy group. Table 3 presents the characteristics of the detected polyps (per-polyp analysis). There were no significant differences in the location, size, morphology, or histological findings of the polyps between the two groups. The mean size of the detected lesions was 4.89 mm (SD, 2.27) in the AI-assisted colonoscopy group and 4.64 mm (SD, 2.46) in the standard colonoscopy group. Approximately 69% of the lesions were diminutive polyps (≤ 5 mm in size). Regarding morphology, 35.2% of the polyps detected in the AI-assisted colonoscopy group and 29.9% in the standard colonoscopy group were flat lesions, a difference that was not statistically significant.

**Table 3:** Characteristics of the detected lesions

		AI group (*n* = 583)	SC group (*n* = 592)	*p*-value
Location				0.151
	Proximal^*^	353 (60.5)	333 (56.2)	
	Distal^†^	230 (39.5)	259 (43.8)	
Size				0.239
	≤ 5 mm	390 (66.9)	422 (71.3)	
	6–9 mm	166 (28.5)	149 (25.2)	
	≥ 10 mm	27 ( 4.6)	21 ( 3.5)	
Morphology				0.062
	Flat	205 (35.2)	177 (29.9)	
	Protruded	378 (64.8)	415 (70.1)	
Histologic findings				0.059
	Adenoma with LGD	326 (55.9)	298 (50.3)	
	Adenoma with HGD or villous adenoma	3 ( 0.5)	12 ( 2.0)	
	Invasive carcinoma	0 ( 0.0)	0 ( 0.0)	
	SSL without dysplasia	24 ( 4.1)	21 ( 3.5)	
	SSL with dysplasia	10 ( 1.7)	8 ( 1.4)	
	Hyperplastic polyp	106 (18.2)	132 (22.3)	
	Traditional serrated adenoma	0 ( 0.0)	2 ( 0.3)	
	Others^‡^	114 (19.6)	119 (20.1)	

Values are presented with numbers (%)

AI, AI-assisted colonoscopy; SC, standard colonoscopy; LGD, low-grade dysplasia; HGD, high-grade dysplasia; SSL, sessile serrated lesion

^*^ Proximal colon was defined as the segment extending from the cecum to the splenic flexure

^†^Distal colon was defined as the segment extending from the descending colon to the rectum

^‡^Others included inflammatory polyps, lymphoid aggregates, and nonspecific findings

### Subgroup analysis categorized by endoscopists

The ADRs of the four experienced endoscopists were 19.0%, 40.7%, 21.4%, and 45.7%, respectively. Based on their ADRs, endoscopists were categorized as high detectors if their ADR was ≥ 25% and low detectors if it was < 25%. Additionally, five endoscopy trainees performed 114 colonoscopies (33 AI-assisted and 81 standard), achieving an overall ADR of 34.2%.

Table 4 presents lesion detection rates categorized by the endoscopists. In endoscopy trainees, ADRs were significantly higher in the AI-assisted colonoscopy group compared to the standard colonoscopy group (51.5% vs. 27.2%, *p* = 0.023). Any polyps and SSLs were detected more frequently in the AI-assisted colonoscopy group, although the difference was not statistically significant. Among the high-detectors, SSL detection rates (SSLDR) were significantly higher in the AI-assisted colonoscopy group compared to the standard colonoscopy group (8.0% vs. 3.3%, *p* = 0.043). The detection rates of any polyps, adenomas, and advanced adenomas were numerically higher in the AI-assisted colonoscopy group, although the differences were not statistically significant. In contrast, detection rates of any lesions did not differ significantly between the two colonoscopy groups among the low-detectors.

**Table 4:** Lesion detection rates categorized by endoscopists

Lesion detection rates of endoscopy trainees (%)				
	Adenomas	AAs^*^	SSLs	Any polyps
AI group (*n* = 33)	51.5	0	6.1	72.7
SC group (*n* = 82)	27.2	3.7	2.5	50.6
*p*-value	0.023	0.555	0.578	0.051
Lesion detection rates of high-detectors^†^ (%)				
	Adenomas	AAs	SSLs	Any polyps
AI group (*n* = 251)	45.8	4.4	8.0	64.9
SC group (*n* = 240)	39.2	3.3	3.3	61.3
*p*-value	0.162	0.713	0.043	0.451
Lesion detection rates of low-detectors^‡^ (%)				
	Adenomas	AAs	SSLs	Any polyps
AI group (*n* = 190)	20.0	2.1	2.1	34.2
SC group (*n* = 290)	19.3	1.4	3.4	32.8
*p*-value	0.945	0.718	0.563	0.817

AI, AI-assisted colonoscopy; SC, standard colonoscopy; AA, advanced adenoma; SSL, sessile serrated lesion

^*^Advanced adenomas were defined as adenomas ≥ 10 mm, and/or with villous histology, and/or with high-grade dysplasia

^†^High-detector was defined as an endoscopist with an adenoma detection rate of ≥ 25%

^‡^Low-detector was defined as an endoscopist with an adenoma detection rate of < 25%

### Predictors of adenoma detection

A logistic regression analysis was performed to identify factors associated with adenoma detection (Table 5). In the multivariate regression analysis, age was positively associated with adenoma detection, while female sex, surveillance colonoscopy, and colonoscopy for symptom evaluation were negatively associated with adenoma detection. An inspection time of ≥ 6 min was strongly associated with higher ADRs compared to an inspection time of < 6 min (OR, 5.689; 95% CI, 3.460–9.353). AI-assisted colonoscopy was also associated with higher ADRs than standard colonoscopy (OR, 1.448; 95% CI, 1.090–1.924).

**Table 5:** Predicting factors associated with adenoma detection

	Univariate analysis		Multivariate analysis	
	OR (95% CI)	*p*-value	OR (95% CI)	*p*-value
Age	1.044 (1.033–1.056)	< 0.001	1.043 (1.031–1.056)	< 0.001
Sex				
Male	1.000		1.000	
Female	0.540 (0.415–0.703)	< 0.001	0.584 (0.436–0.782)	< 0.001
BMI	1.051 (1.015–1.089)	0.005	1.021 (0.982–1.063)	0.294
Indication				
Screening	1.000		1.000	
Surveillance Symptomatic	0.802 (0.555–1.161) 0.282 (0.179–0.445)	0.243 < 0.001	0.616 (0.414–0.918) 0.303 (0.186–0.492)	0.017 < 0.001
Bowel preparation (BBPS)	0.894 (0.747–1.072)	0.226	1.010 (0.828–1.231)	0.923
Inspection time				< 0.001
< 6 min	1.000		1.000	
≥ 6 min	6.602 (4.389–9.931)	< 0.001	5.689 (3.460–9.353)	
Colonoscopy				
Standard	1.000		1.000	
AI-assisted	1.427 (1.103–1.847)	0.007	1.448 (1.090–1.924)	0.011

OR, odds ratio; CI, confidence interval; BMI, body mass index; BBPS, Boston Bowel Preparation Scale

## Discussion

This study aimed to evaluate the effectiveness of AI-assisted colonoscopy in a real-world setting. AI-assisted colonoscopy resulted in significantly higher ADRs and APCs compared to standard colonoscopy (ADR, 35.9% vs. 26.4%; APC, 0.69 vs. 0.43). However, the detection rates of advanced adenomas and SSLs did not significantly differ between the two groups.

Multiple RCTs have evaluated the effectiveness of AI-assisted colonoscopy, and a recent meta-analysis of 21 RCTs confirmed that AI-assisted colonoscopy increases the ADR [18]. However, two main issues limit the applicability of these findings in real-world clinical practice. First, the lack of blinding may have introduced a bias favoring the AI group. Although one double-blind RCT using a sham control showed increased ADR [9], there remains a lack of sufficient blinded studies. Second, these RCTs were conducted under strict conditions that may not reflect real-world factors affecting colonoscopy quality, such as examiner fatigue or time pressure. To address this, non-RCT observational studies have been conducted in real-world clinical practice settings. A meta-analysis by Patel et al. found no significant improvement in polyp detection using AI in real-world settings [13], while another systematic review reported a small but statistically significant improvement in ADR (36.3% vs. 35.8%, *p* = 0.04) [14]. Considering the inconsistent outcomes of previous non-RCTs, this study provides substantial evidence supporting the role of AI in clinical practice, demonstrating an increase in ADR in the AI-assisted colonoscopy group. Several other studies using AI-assisted colonoscopy support our findings. For instance, a single-center retrospective study showed a significant improvement in ADR with AI assistance compared to conventional methods (47.9% vs. 38.5%, *p* = 0.03) [19]. Similarly, a study from Korea found a significantly higher ADR in the AI-assisted group compared to the standard colonoscopy group (45.1% vs. 38.8%, *p* = 0.010) [20]. An additional finding in our study was that the overall SSLDR increased from 3.0 to 5.5%, although the difference was not statistically significant (*p* = 0.076), likely due to the limited sample size. However, considering that the SSLDR in studies without CADe systems typically ranges from 2 to 2.5% [21, 22], our finding is noteworthy. Similar to our results, a recent study using the CADe system reported an increase in the SSLDR from 2.5 to 5.7% (*p* = 0.001) [20], although another study did not show significant changes [19], indicating ongoing controversy. Unlike ADR, there are no universally accepted benchmarks for SSLDR, despite SSLs accounting for 15–30% of CRC cases [23]. Due to their flat morphology, SSLs are particularly challenging to detect, highlighting the need for further investigation into the role of AI in improving SSLDR.

In contrast, other studies have reported that AI-assisted colonoscopy does not significantly improve ADR in real-world settings [10–12]. Although our study showed a positive effect of AI-assisted colonoscopy on ADR, this result may have been influenced by the fact that the endoscopists were aware that their colonoscopy performance was being monitored, potentially leading to changes in their behavior. Moreover, more than two-thirds of the detected lesions were < 5 mm in size. Therefore, the increased ADR associated with AI-assisted colonoscopy may result from the increased detection of diminutive adenomas. Considering that a long-term prospective study showed that most diminutive polyps exhibit slow growth and typically follow a benign course [24], this raises questions regarding their clinical relevance. This finding emphasizes the need for a long-term follow-up study to clarify the effect of AI-assisted colonoscopy on the prevention of CRC. Moreover, there was no significant difference in advanced adenoma detection between the AI-assisted colonoscopy and standard colonoscopy groups, likely because these lesions are easier to detect without AI assistance. Another limitation of CADe is the frequent occurrence of false positives due to mucosal folds or residuals, which can lead to unnecessary resections, longer procedure times, and increased costs.

Although CADe is a valuable tool in colonoscopy, the role of endoscopists remains crucial, as polyp detection depends on their ability to ensure adequate mucosal exposure and identify abnormalities [25]. Subgroup analysis in this study showed no significant difference in ADRs between AI-assisted colonoscopy and standard colonoscopy in the low-detector group (20% vs. 19.3%, *p* = 0.945). In contrast, the high-detector group exhibited a numerically higher ADR (45.8% vs. 39.2%, *p* = 0.162) and a statistically significant increase in SSLDR (8.0% vs. 3.3%, *p* = 0.043) with AI-assisted colonoscopy. This suggests that the additional benefits of CADe with increased ADRs may be limited if the endoscopist does not perform a meticulous examination. However, the observed differences in ADR according to endoscopist performance in our study were based on only two endoscopists per group, providing insufficient evidence to support the generalization of this finding and thus should be interpreted with caution. Further large-scale studies involving more endoscopists are necessary to confirm and validate this observation. Regarding endoscopy experience, CADe was associated with increased ADR in the trainee group (51.5% vs. 27.2%, *p* = 0.023) in the subgroup analysis, although the small sample size may have affected the reliability of the results. A previous study indicated that CADe helps trainees improve their ADR, thereby reducing the performance gap between trainees and experts [26]. This suggests that AI can reduce the learning curve of trainees and lead to better screening outcomes, as it can assist trainees in lesion recognition by pointing out areas that might otherwise be overlooked by trainees during the procedure. Further studies are needed to assess the impact of AI-assisted colonoscopy across endoscopists with varying levels of experience to better understand its effects on detection and screening outcomes.

A limitation of AI-assisted colonoscopy is its inability to detect lesions in areas that are not fully exposed. Various mucosal exposure devices, such as transparent caps and Endocuff Vision, have been extensively studied for their efficacy. The Endocuff, a device designed to flatten colon folds and improve mucosal visibility, has demonstrated efficacy in several RCTs, significantly improving ADR compared to standard colonoscopy [27]. A study comparing colonoscopy with the combination of CADe and Endocuff to CADe alone found that the ADR was significantly higher in the Endocuff combination group [28]. Additionally, a recent three-arm RCT demonstrated that combining Endocuff with CADe significantly improved the ADR (58.7%) compared with CADe alone (53.8%) or standard colonoscopy (46.3%) [29]. However, the effectiveness of transparent caps in improving ADR remains controversial, with some studies showing benefits and others showing no significant effect [30]. In our study, the use of transparent caps was based on the endoscopists’ preferences, making it challenging to assess the specific effects of their combination with CADe. Further research is needed to determine the optimal combination of mucosal exposure devices and CADe to enhance the polyp detection outcomes.

This study’s non-randomized observational design enhances the applicability of its findings to real-world clinical practice. Additionally, propensity score matching reduced group bias, improving the reliability of our findings. Baseline characteristics such as age, sex, and BMI showed no significant differences, and these were further minimized in the matched cohort, indicating an effective bias control. Using a concurrent control group was another strength of this study. While historical controls are susceptible to various biases, concurrent control groups minimize differences caused by time-related changes and external factors, thereby providing greater validity.

This study had some limitations. First, it was conducted at a single center, limiting the generalizability of the results and emphasizing the need for multicenter studies to improve external validity. In addition, since this was a non-blinded study, there is a possibility of selection bias in patient allocation. However, we minimized this potential bias by applying propensity score matching to adjust for baseline characteristics between the two groups. Second, the relatively small number of endoscopists, with two of the four endoscopists having an ADR < 25%, may have influenced the findings. Including endoscopists with low baseline ADRs may have contributed to the overall increase in the ADR observed in this study. Future studies adjusting for endoscopists’ baseline ADRs will provide a clearer assessment of the true impact of AI. Third, the endoscopists’ awareness of AI usage may have prompted more careful evaluations, potentially influencing the results. Implementing a blinded design may yield more relevant findings in future studies. Furthermore, the study excluded patients with complicated colonic conditions, such as inflammatory bowel disease or polyposis syndrome, limiting the applicability of the findings to these populations. Therefore, future studies should include a broader range of patients. Moreover, the study did not establish a connection between improved ADRs and long-term patient benefits, such as reduced CRC incidence, highlighting the need for further research to evaluate these long-term outcomes. Finally, we did not perform a separate analysis of detection rates for laterally spreading tumors (LST), particularly the non-granular (LST-NG) subtype. These lesions are known to be easily missed and more difficult to achieve complete resection, making them clinically significant [31]. Therefore, the detection rate of LSTs should be considered an important component in evaluating the performance of CADe systems, underscoring the need for future large-scale studies to address this issue.

In conclusion, AI-assisted colonoscopy significantly improved ADR in real-world settings, demonstrating its potential to enhance screening quality and facilitate early polyp detection. The synergy between CADe and the endoscopist’s careful observation offers promising advancements in CRC prevention.

## Data Availability

The data support the findings of this study and are available within the article.

## Abbreviations

ADR: Adenoma Detection Rate
AI: Artificial Intelligence
APC: Adenomas per Colonoscopy
BBPS: Boston Bowel Preparation Scale
BMI: Body Mass Index
CADe: Computer-Aided Detection
CI: Confidence Interval
CRC: Colorectal Cancer
FPN: Feature Pyramid Network
HGD: High-Grade Dysplasia
LGD: Low-Grade Dysplasia
LST: Laterally Spreading Tumors
OR: Odds Ratio
PDR: Polyp Detection Rate
RCT: Randomized Controlled Trial
SC: Standard Colonoscopy
SD: Standard Deviation
SSL: Sessile Serrated Lesion
SSLDR: Sessile Serrated Lesion Detection Rate
